# Transcatheter closure of a ruptured sinus of Valsalva aneurysm—a minimally invasive approach to a rare cardiac emergency: a case report

**DOI:** 10.1093/ehjcr/ytag130

**Published:** 2026-03-02

**Authors:** Bahram Shahri, Mohsen Moohebati, Mostafa Ahmadi, Mahdi Kahrom, Mohammad Hussein Hasin

**Affiliations:** Department of Cardiovascular Diseases, Faculty of Medicine, Mashhad University of Medical Sciences, Ghaem Hospital, Ahmadabad Blvd, Mashhad 9176699199, Iran; Heart and Vascular Research Center, Mashhad University of Medical Sciences, Ghaem Hospital, Ahmadabad Blvd, Mashhad 9176699199, Iran; Atherosclerosis Prevention Research Center, Faculty of Medicine, Mashhad University of Medical Sciences, Ghaem Hospital, Ahmadabad Blvd, Mashhad 9176699199, Iran; Department of Cardiovascular Surgery, Faculty of Medicine, Mashhad University of Medical Sciences, Ghaem Hospital, Ahmadabad Blvd, Mashhad 9176699199, Iran; Interventional Cardiologist, Mashhad University of Medical Sciences, Ghaem Hospital, Ahmadabad Blvd, Mashhad 9176699199, Iran

**Keywords:** Sinus of Valsalva aneurysm, Ruptured aneurysm, Transcatheter closure, Aorto-right ventricular fistula, Structural heart disease, Case report

## Abstract

**Background:**

Sinus of Valsalva aneurysm (SVA) is a rare but clinically significant cardiac anomaly, arising congenitally or through acquired causes. Typically, asymptomatic until rupture, this condition can precipitate acute or subacute heart failure via a pathological left-to-right shunt. While surgical repair has long been considered the definitive treatment, advances in high-resolution imaging and percutaneous device technology have established transcatheter closure as a compelling, minimally invasive alternative in anatomically suitable patients.

**Case summary:**

We present a case of a ruptured noncoronary sinus of Valsalva aneurysm creating a left-to-right shunt into the right ventricle. Despite the potentially life-threatening haemodynamic implications, the patient maintained stability. Multimodal imaging—including transoesophageal echocardiography and computed tomography angiography—precisely delineated the defect and informed procedural strategy. The patient underwent a successful percutaneous transcatheter closure using a patent ductus arteriosus occluder device, with no procedural complications. Follow-up evaluation confirmed complete defect occlusion and preservation of aortic valve integrity, with no residual shunting.

**Conclusion:**

This case underscores the expanding role of transcatheter interventions in the management of ruptured SVAs, highlighting the importance of meticulous imaging and patient selection to achieve excellent clinical and structural outcomes. It contributes to the growing evidence supporting minimally invasive alternatives to surgery in selected structural heart diseases.

Learning points

- Transcatheter closure of ruptured sinus of Valsalva aneurysm is a viable, life-saving alternative to surgery in anatomically suitable patients, offering rapid stabilization with minimal invasiveness.
- This case highlights the paradigm shift towards percutaneous solutions in structural cardiac emergencies, underscoring the importance of timely diagnosis and individualized intervention.
- A ruptured sinus of Valsalva should be on the differential diagnosis in patients with sudden heart failure and a new continuous murmur, even in the absence of prior cardiovascular disease—early recognition is critical to survival.

## Introduction

Sinus of Valsalva aneurysm (SVA) is a rare yet potentially catastrophic cardiac anomaly, accounting for less than 1% of congenital heart defects. It arises from structural weakness at the junction of the aortic media and the annulus fibrosus, most commonly involving the right or noncoronary sinus. While many SVAs remain clinically silent, rupture into adjacent cardiac chambers—most frequently the right atrium or right ventricle—can trigger abrupt haemodynamic compromise due to the creation of a continuous left-to-right shunt. The clinical spectrum ranges from asymptomatic murmurs to fulminant heart failure, often masquerading as more common valvular or shunt pathologies.

Historically, surgical repair has served as the gold standard for the treatment of ruptured SVAs, offering durable results but at the cost of cardiopulmonary bypass and thoracotomy-associated morbidity. However, in recent years, the landscape of structural heart intervention has undergone a transformative shift. The maturation of transcatheter closure technologies—particularly the use of occluder devices originally designed for patent ductus arteriosus and ventricular septal defects—has enabled experienced operators to manage selected cases of RSOV aneurysm with precision, efficacy, and minimal invasiveness. Leveraging advanced imaging modalities such as transoesophageal echocardiography and high-resolution cardiac CT, transcatheter intervention allows for real-time anatomical navigation, defect sizing, and device deployment, even in complex aorto-cardiac communications.

Despite these advances, transcatheter closure of ruptured SVA remains underreported in the literature worldwide. Most involve rupture into the right heart chambers, and the anatomical diversity of these lesions necessitates individualized procedural planning. In this context, we present the case of a 44-year-old woman with a ruptured noncoronary SVA into the right ventricle, who was treated successfully using a percutaneous transcatheter approach. This case not only exemplifies the diagnostic value of multimodality imaging but also reinforces the feasibility and safety of catheter-based closure in anatomically favourable presentations. It adds to the growing body of evidence supporting transcatheter therapy as a definitive alternative to surgery in selected patients with isolated SVA rupture.

## Summary figure

**Figure:**
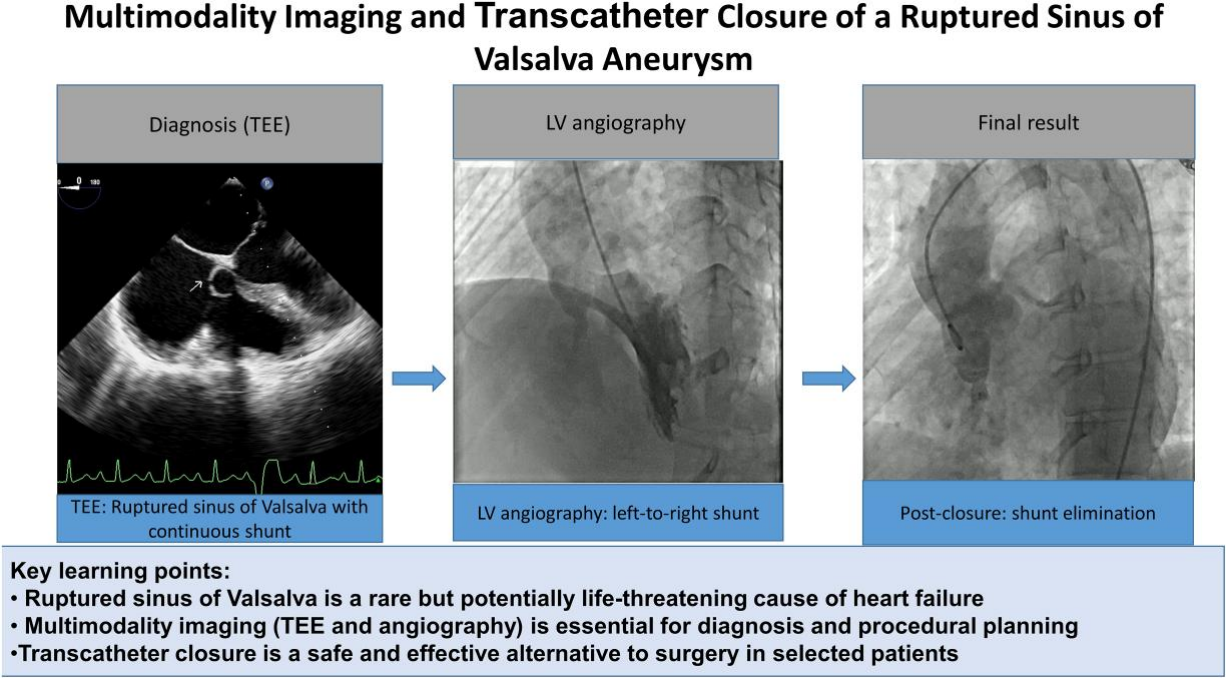
Multimodality imaging and percutaneous management of a ruptured sinus of Valsalva aneurysm. Transoesophageal echocardiography confirms rupture with left-to-right shunt into the right ventricle. Angiography demonstrates the fistulous communication. Final fluoroscopic imaging shows successful transcatheter device closure with elimination of the shunt.

## Case presentation

A 44-year-old woman with a medical history of hypertension and type 2 diabetes mellitus presented with progressive exertional dyspnoea over 6 months, which had significantly worsened during the preceding 3 months. She denied chest pain, palpitations, syncope, orthopnea, or fever. There was no prior history of congenital heart disease, rheumatic fever, endocarditis, or cardiothoracic surgery.

On physical examination, the patient was haemodynamically stable and afebrile. Auscultation revealed a high-pitched, continuous murmur best heard at the left parasternal border in the third intercostal space. No peripheral oedema, jugular venous distention, or hepatomegaly was noted. Her functional capacity was consistent with New York Heart Association (NYHA) class III.

**Figure 1:**
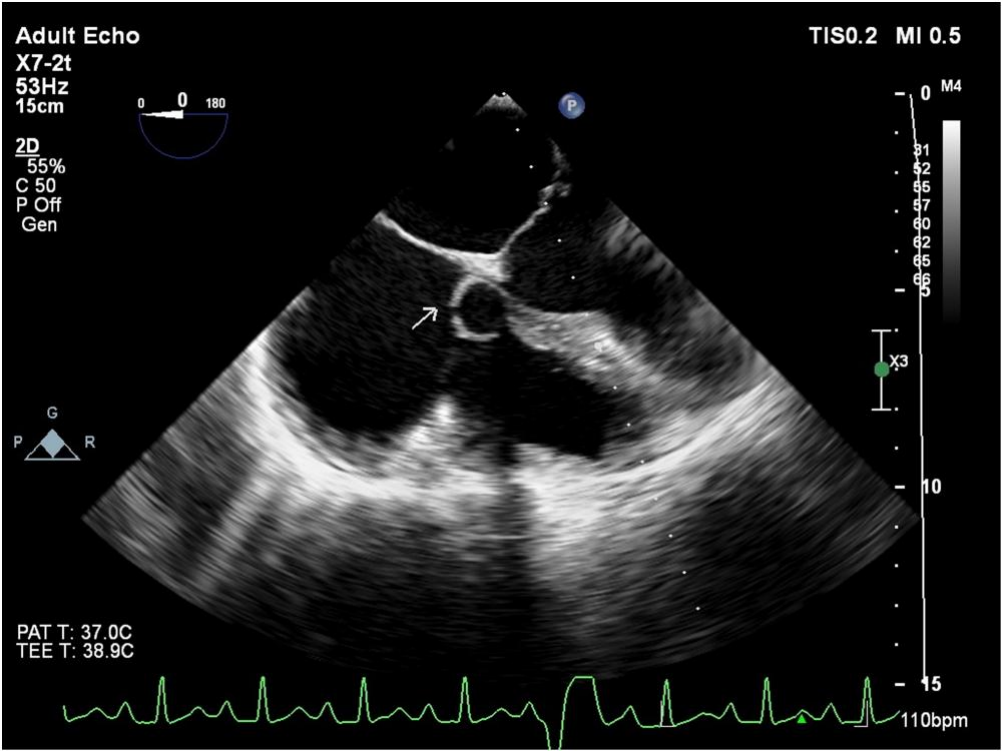
Transoesophageal echocardiography identifying ruptured sinus of Valsalva. Mid-esophageal transoesophageal echocardiography (TEE) short-axis view illustrates an aneurysmal pouch arising from the aortic root that communicates with the right-sided cardiac chamber, consistent with a rupture sinus of Valsalva. The anatomy of the aortic root is well delineated, facilitating confirmation of rupture site and procedural planning. Abbreviations: TEE, transoesophageal echocardiography.

Transthoracic echocardiography (TTE) demonstrated mild enlargement of the left ventricle with preserved systolic function, corresponding to a left ventricular ejection fraction of approximately 55%–60%. The right ventricle was markedly dilated but maintained preserved systolic function. Both atria were enlarged. No pericardial effusion or additional structural abnormalities were observed. Transoesophageal echocardiography (TEE) further delineated a large saccular aneurysm of the noncoronary sinus of Valsalva measuring approximately 36 mm × 39 mm, with a 7 mm rupture into the right ventricle. Continuous-wave Doppler revealed a high-velocity, continuous left-to-right shunt from the aneurysmal sac into the right ventricle. Mild to moderate aortic regurgitation was present without cusp prolapse or leaflet disruption (*Figure 1*). No vegetations, intracardiac thrombus, or pericardial effusion were noted. The detailed anatomic assessment provided by TEE facilitated precise procedural planning for transcatheter closure.

Computed tomography angiography (CTA) confirmed the presence of a saccular aneurysm originating from the noncoronary sinus, projecting inferiorly towards the right ventricle. A tubular, tapering fistulous tract was identified, measuring 12 mm at its aortic origin and narrowing to 7 mm distally. The aortic root, ascending aorta, and pulmonary artery were otherwise unremarkable. No evidence of dissection, thrombus, or pericardial effusion was seen.

**Figure 2:**
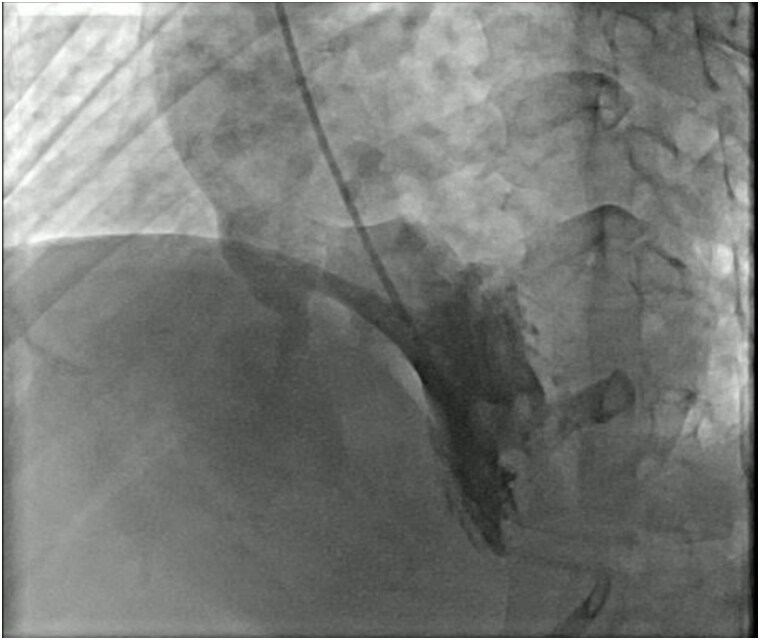
Left ventriculogram demonstrating ruptured sinus of Valsalva with left-to-right shunt. Left ventriculography reveals contrast passage from the left ventricular outflow tract into the right ventricle, indicating a ruptured sinus of Valsalva aneurysm resulting in a continuous left-to-right shunt. The catheter tip is visible within the left ventricle (LV), and contrast opacifies the right ventricle (RV). Abbreviations: LV, left ventricle; RV, right ventricle.

Coronary angiography demonstrated minimal nonobstructive coronary artery disease. Aortic root injection clearly visualized contrast opacification of the right ventricle during systole and diastole, confirming the presence of a significant left-to-right shunt through the ruptured sinus of Valsalva (*Figure 2*). Right ventricular dilation was also noted fluoroscopically.

Intervention for ruptured sinus of Valsalva aneurysms is guided by the haemodynamic impact of the shunt, right heart remodelling, and the presence of progressive heart failure. In this patient, the continuous left-to-right shunt, severe right ventricular dilation, and worsening exertional symptoms indicated the need for closure. Considering the aneurysm’s favourable anatomy and the patient’s preserved hemodynamics, a percutaneous transcatheter approach was selected as the preferred treatment strategy, enabling safe and effective closure as a minimally invasive alternative to surgery. This case highlights the importance of individualized clinical assessment in managing rare structural cardiac anomalies.

**Figure 3:**
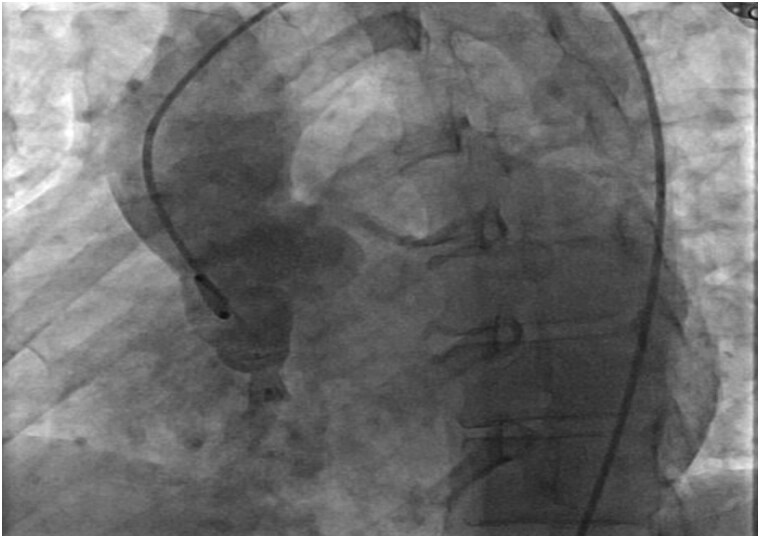
Postprocedural angiogram confirming device closure of ruptured sinus of Valsalva. Final fluoroscopic angiographic frame demonstrates correct deployment of an occluder device at the aneurysm neck, with preservation of aortic root integrity and no residual contrast leakage, indicating successful closure of the ruptured sinus tract. The catheter and device are clearly visualized.

After careful discussion within the multidisciplinary cardiac team, including interventional cardiology and cardiac surgery, and taking into account the patient’s preference, percutaneous transcatheter closure was selected. The aneurysm’s favourable anatomy and the patient’s preserved hemodynamics supported this minimally invasive approach as a safe and effective alternative to surgery. The intervention was performed via femoral arterial and venous access using 6 Fr sheaths. A 0.035-inch J-tipped guidewire was advanced retrogradely from the aortic root through the ruptured noncoronary sinus of Valsalva into the right ventricle and snared within the femoral venous sheath, thereby establishing a stable arteriovenous loop. Over this rail, an 8 mm × 10 mm patent ductus arteriosus occluder device (Cocoon, Vascular Innovations Co.) was carefully advanced and deployed across the defect under fluoroscopic guidance. Proper device positioning was confirmed angiographically, demonstrating complete closure of the fistulous tract with no residual shunt (*Figure 3*). Aortic valve integrity was preserved, and the procedure was well tolerated, underscoring that a percutaneous approach can provide safe and effective closure in anatomically suitable ruptured sinus of Valsalva aneurysms.

The patient had an uneventful recovery and was discharged on single antiplatelet therapy with aspirin for a planned duration of 6 months, consistent with common practice following structural device implantation. At 1-month follow-up, she remained asymptomatic and had returned to New York Heart Association functional class I. Follow-up transthoracic echocardiography confirmed persistent closure of the fistula, with no residual shunt, preserved biventricular function, and stable valvular findings. Given the patient’s age and the rarity of this condition, ongoing clinical and imaging surveillance is warranted to ensure durable device stability, detect any potential late-onset haemodynamic or valvular changes, and guide long-term management. Follow-up will be tailored according to clinical findings and established practice in similar structural heart interventions.

## Discussion

Sinus of Valsalva aneurysm (SVA) represents a rare but clinically significant cardiovascular abnormality, most often congenital in origin due to focal discontinuity or structural weakness within the elastic lamina of the aortic media. Less commonly, it may arise as an acquired lesion secondary to infective, traumatic, or degenerative processes. While most SVAs are isolated, rare associations with connective tissue disorders or other congenital cardiac anomalies have been reported. In the absence of additional syndromic or structural findings, these aneurysms are typically sporadic, and routine genetic testing or family screening is not indicated. Nonetheless, recognition of these potential associations is important for comprehensive patient counselling and for guiding individualized clinical management in such rare and complex cases.^1^ In its unruptured state, SVA typically remains clinically silent and is often detected incidentally. However, rupture transforms this quiescent lesion into a surgical or interventional emergency, producing a sudden or progressive left-to-right intracardiac shunt—most frequently into the right atrium or right ventricle—resulting in volume overload and acute heart failure.^2^ Among the three sinuses, the right coronary sinus is the most commonly affected; rupture originating from the noncoronary sinus is exceedingly uncommon, thereby rendering our case not only diagnostically challenging but also of notable rarity within the published literature.^1,3^

The classical clinical presentation of ruptured SVA includes exertional dyspnoea, continuous machinery murmur, and signs of volume overload, consistent with our patient's symptoms and physical findings.^4^ Multimodality imaging is essential for accurate diagnosis and procedural planning. Transoesophageal echocardiography (TEE) remains the cornerstone for defining the anatomy and detecting the shunt.^5^ Coronary computed tomography angiography (CTA) provides detailed anatomic visualization of the aneurysm, its size, orientation, and the fistulous tract, while coronary angiography is critical to exclude significant concomitant coronary artery disease and to confirm the shunt hemodynamics.^6^ Our case underscores the utility of integrating these modalities to precisely delineate the anatomy and guide therapeutic decisions.

Historically, surgical repair has been the definitive treatment for ruptured SVA, offering excellent long-term outcomes but associated with the inherent risks of open heart surgery, including morbidity from cardiopulmonary bypass and prolonged recovery.^7^ Over the past two decades, transcatheter closure has emerged as a less invasive and increasingly preferred alternative in anatomically suitable patients, particularly those who are haemodynamically stable.^8^ Limited cases have been reported utilizing percutaneous closure devices, with the majority involving the right coronary sinus.^3^ Our case adds to this growing body of evidence by demonstrating successful closure of a ruptured noncoronary sinus aneurysm using a patent ductus arteriosus (PDA) occluder device.

Device selection is pivotal, with PDA occluders favoured for their shape and flexibility that accommodate the fistulous anatomy.^9^ Although most reported interventions have used combined fluoroscopic and echocardiographic guidance, our successful deployment under fluoroscopy alone highlights the procedural adaptability, which may be advantageous in resource-limited settings or when echocardiographic windows are suboptimal.^3^ The absence of residual shunting or valvular dysfunction at one month follow-up reflects the safety and efficacy of this approach.

Nonetheless, long-term data on transcatheter closure of ruptured SVAs remain limited. Potential complications include device embolization, residual shunts, and progressive aortic or tricuspid valve insufficiency, necessitating vigilant follow-up.^10^ Our case emphasizes the importance of individualized patient assessment and tailored intervention strategies based on comprehensive imaging and clinical status.

In conclusion, this case reinforces the feasibility, safety, and effectiveness of percutaneous transcatheter closure as a minimally invasive alternative to surgery in ruptured noncoronary sinus of Valsalva aneurysm. It underscores the expanding role of catheter-based therapies in managing rare structural cardiac anomalies and highlights the critical importance of multimodality imaging in diagnosis and treatment planning.

## Conclusion

This case exemplifies the evolving role of transcatheter techniques in the management of rare structural heart defects. The successful percutaneous closure of a ruptured noncoronary sinus of Valsalva aneurysm—performed solely under fluoroscopic guidance using a PDA occluder device—demonstrates the feasibility, safety, and clinical efficacy of catheter-based intervention in carefully selected patients. As imaging technologies and device designs continue to advance, transcatheter closure offers a minimally invasive, patient-tailored alternative to surgical repair, expanding therapeutic options for conditions once considered operable only through open-heart surgery.

## Supplementary Material

ytag130_Supplementary_Data

## Data Availability

All relevant data supporting the findings of this case report are included within the article and its supplementary materials. Additional data are available from the corresponding author upon request.

## Lead author biography

Bahram Shahri serves as an accomplished interventional cardiologist within the Department of Cardiovascular Diseases at the Faculty of Medicine, Mashhad University of Medical Sciences, Mashhad, Iran. He practices at one of the region=E2=80=99s foremost academic hospitals, where he is actively engaged in the clinical training of cardiology residents. Dr. Shahri has contributed extensively to the field through numerous scholarly investigations and remains committed to advancing the science and practice of interventional cardiology. His academic interests lie in complex coronary interventions and the development of minimally invasive therapeutic strategies in cardiovascular medicine.
